# Next‐Generation Vaccines for Co‐Circulating PEDV and TGEV: Integrating Nucleic Acid Platforms, Mucosal Delivery, and AI–Driven Antigen Design

**DOI:** 10.1155/tbed/2014296

**Published:** 2025-12-04

**Authors:** Xiaojun Hu, Zhenshan Wang, Shen Wang, Hongyu Sun, Na Feng, Entao Li, Xianzhu Xia, Guixue Hu, Feihu Yan

**Affiliations:** ^1^ College of Veterinary Medicine, Jilin Agricultural University, Changchun, 130118, Jilin, China, jlau.edu.cn; ^2^ Changchun Veterinary Research Institute, Chinese Academy of Agricultural Sciences, State Key Laboratory of Pathogen and Biosecurity, Key Laboratory of Jilin Province for Zoonosis Prevention and Control, Changchun, 130000, China, caas.cn; ^3^ Key Laboratory of Special Animal Epidemic Disease, Ministry of Agriculture, Jilin Provincial International Cooperation Key Laboratory for Science and Technology Innovation of Special Animal and Plants, Institute of Special Institute of Special Animal and Plant Sciences, Chinese Academy of Agricultural Sciences, Changchun, 130112, China, caas.cn; ^4^ Center for Infectious Medicine and Vaccine Research, School of Basic Medicine and Clinical Pharmacy, China Pharmaceutical University, Nanjing, 211198, China, cpu.edu.cn; ^5^ Innovation Center for Nucleic Acid Medicine, Institute for Innovative Drug Development and Life Sciences, Wuxi, 214000, China

**Keywords:** AI–assisted antigen design, mucosal immunity, PEDV, TGEV, vaccine

## Abstract

Porcine epidemic diarrhea virus (PEDV) and transmissible gastroenteritis virus (TGEV) are causative agents of acute enteric diseases in pigs and have a high contagion potential. These coronaviruses (CoVs) impose substantial economic losses on global pork production, particularly affecting lactating piglets where coinfections occur. Although traditional vaccines offer partial protection, their efficacy is increasingly challenged by the continuous emergence of mutated strains of PEDV and TGEV. This underscores the demand for novel vaccines with improved protective efficacy and cost‐effectiveness. Emerging vaccine technologies, such as nucleic acid vaccines, genetically engineered subunit vaccines, and live vector vaccines, have received widespread attention because of their advantages in terms of safety, stability, targeted delivery, economy, and ease of use. This review summarizes recent advances in PEDV and TGEV vaccine development, highlighting both their potential and limitations. More importantly, we prospect novel techniques that may supplement the status gaps and lead to breakthroughs in blocking the transmission of these CoVs. Notable research priorities encompass mucosal immunity mechanisms, vertical transmission prevention strategies, and computational immunogen design leveraging artificial intelligence (AI). Overall, a deeper understanding of the pathogens coupled with technological advances is expected to accelerate the control of and effective response to pathogenic CoVs, thereby safeguarding the stability of animal husbandry.

## 1. Introduction

Coronaviruses (CoVs) represent a group of single‐stranded positive‐sense RNA viruses characterized by genomic lengths ranging from 26 to 32 kilobases (kb; Figure 1) [1, 2], encompassing multiple porcine pathogens of clinical significance. Within the *Alphacoronavirus* genus (family Coronaviridae), porcine epidemic diarrhea virus (PEDV) and transmissible gastroenteritis virus (TGEV) have substantial epidemiological importance because of their ability to provoke acute and highly contagious enteropathogenic diarrhea in swine populations [3]. Although these viruses exhibit distinct genetic and antigenic profiles, they present overlapping clinical presentations, including vomiting, watery diarrhea, and dehydration, with mortality rates as high as 95% in neonatal piglets [4–6].

Figure 1Diagram of the viral genome structure and replication cycle [1]. (A) The diagram depicts a coronavirus particle’s structural proteins (S, E, M, and N) and RNA genome, alongside genomic comparisons of PEDV and TGEV, highlighting conserved 5′UTRs, ORF1a/1b, and structural gene regions. (B) The replication cycle outlines key stages: receptor binding/entry, genome release, protein translation, RNA replication, nucleocapsid assembly, virion formation, and ER–mediated budding, elucidating coronavirus propagation mechanisms.

**Figure:**
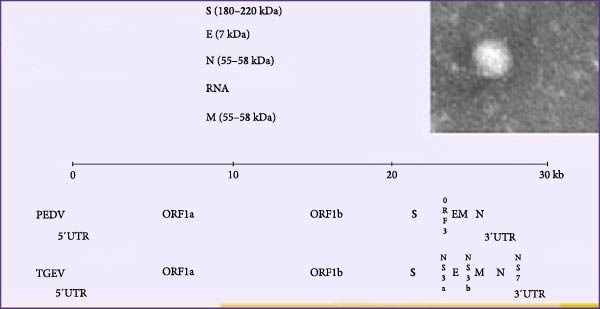
(A)

**Figure:**
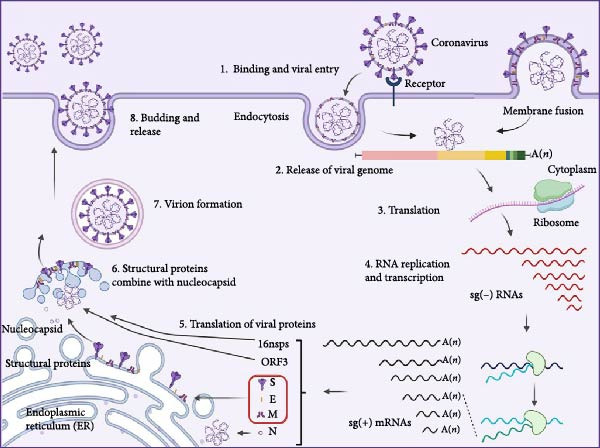
(B)

According to historical surveillance records, TGEV was first identified in the United States in 1946, followed by the detection of PEDV in the United Kingdom in 1971. Since then, both viruses have become globally distributed. A striking example of their impact is the widespread PEDV epizootic that occurred across 29 Chinese provinces from 2010 to 2021, which was reflected in a 61.8% infection rate among diarrheic swine specimens. Molecular epidemiological investigations have identified chimeric CoVs in European swine populations, featuring recombinant genomes that combine TGEV structural backbones with PEDV spike (S) glycoprotein genes, highlighting their potential for natural recombination [7–9]. However, substantial phylogenetic divergence between these viruses precludes cross‐protective immune responses [10]. Transmission dynamics exhibit seasonal periodicity, with peak prevalence during spring and winter, thereby complicating disease containment strategies [5]. Although all porcine age cohorts demonstrate susceptibility, lactating piglets display heightened vulnerability to severe clinical outcomes. Concurrent infections with both pathogens can increase morbidity through synergistic pathogenic mechanisms [11]. The global swine industry sustains significant economic losses from these agents, exemplified by the designation of PEDV as a predominant Class II notifiable animal disease in China in 2023 [12].

While commercially approved inactivated and live‐attenuated formulations demonstrate partial protection in neonatal piglets, their effectiveness against currently circulating viral variants remains suboptimal, as evidenced by persistent outbreaks in vaccinated populations [13]. This efficacy gap is exacerbated by high viral mutation rates and ongoing genetic diversification of field strains through recombination events. These limitations highlight the need for innovative vaccine platforms that integrate advanced biotechnological strategies. Next‐generation candidates under development focus on several synergistic approaches: mRNA–based antigen design enabling rapid adaptation to emerging S protein mutations; virus‐like particle (VLP) formulations engineered to increase the conformational immunogenicity of neutralizing epitopes; artificial intelligence (AI)–driven prediction of conserved cross‐reactive epitopes to facilitate multivalent vaccine architectures; and mucosal delivery systems optimized for intestinal immune priming, including nanoparticle (NP) carriers and probiotic‐vectored antigens. Such platforms aim to address critical shortcomings in current vaccines, particularly the inability to induce robust mucosal IgA responses and durable cross‐protective immunity against heterologous strains. The accelerated evolution of PEDV and TGEV underscores the necessity for modular vaccine technologies capable of swift antigenic updates, paralleling advancements in human RNA virus vaccinology. Furthermore, the combined application of structural vaccinology, computational immunology, and targeted immune activation at intestinal sites of viral entry represents a paradigm shift in combating these enteropathogenic CoVs. The implementation of these strategies could substantially mitigate the economic and production impacts sustained by global swine industries while reducing reliance on passive immunization protocols.

This review systematically delineates the research progress on PEDV and TGEV vaccines and highlights their strengths and limitations. Moreover, we pay particular attention to cutting‐edge technological breakthroughs, aiming to provide theoretical foundations and technical road maps for developing next‐generation vaccines and improving preparedness against emerging pathogenic CoVs. For existing vaccine platforms, effectiveness is primarily limited by several common challenges: the inability to elicit robust mucosal immunity at the primary intestinal infection site, a short duration of protection, and inadequate broad cross‐protection against continually evolving heterologous strains. Addressing these limitations is a crucial objective for next‐generation vaccine development.

## 2. Virion Structures and Epidemiological Landscapes of PEDV and TGEV

### 2.1. Genome and Replication Cycle of PEDV and TGEV

CoVs are enveloped and spherical virions (80–120 nm) structurally organized into four conserved proteins: the S glycoprotein, which mediates receptor binding and entry; the membrane (M) protein, which stabilizes the viral envelope; the envelope (E) protein, which facilitates virion assembly; the nucleocapsid (N) protein, which encapsulates the genomic RNA [14, 15].

In PEDV and TGEV, these structural proteins exhibit conserved architectures with functional variations critical to pathogenesis. Both viruses feature an S protein divided into S1 and S2 subunits: PEDV S1 contains defined receptor‐binding domains (RBDs; S1‐N‐terminal domain [NTD]/S1‐CTD), while its S2 subunit mediates membrane fusion via heptapeptide repeats (HR1/HR2) [16]. In contrast, the TGEV S protein relies on acid‐dependent fusion through less‐characterized peplomers, despite shared roles in receptor‐mediated entry and tissue tropism. The PEDV S protein further distinguishes itself by the presence of four vaccine‐targeted B‐cell epitopes (COE, SS2, SS6, and 2C10), which are absent in TGEV [17]. Structural conservation of the E protein across both viruses masks functional divergence—PEDV E modulates retinoic acid–inducible gene I (RIG‐I)–mediated immunity and induces membrane curvature, whereas the function of TGEV E remains ambiguous beyond its role in assembly [18]. Similarly, while M proteins in both CoVs coordinate virion assembly through nucleocapsid interactions, PEDV M uniquely suppresses host immunity via interferon‐beta (IFN‐*β*) inhibition and S‐phase arrest [19, 20]. The conserved role of the N protein in RNA chaperoning is augmented in PEDV by pleiotropic immunomodulation, including IFN Regulatory Factor 3 (IRF3) suppression and endoplasmic reticulum (ER) stress induction, alongside the enhancement of related viruses such as porcine reproductive and respiratory syndrome virus (PRRSV) [21, 22]. These functional adaptations reflect evolutionary divergence, with PEDV employing specialized mechanisms (e.g., open reading frame 3 (ORF3) ion channel activity) absent in TGEV, yet both viruses converge on ER–Golgi intermediate compartment (ERGIC)–mediated virion maturation and secretion [23, 24].

Following host cell entry, the coronavirus replication cycle begins with cytoplasmic uncoating, which releases the positive‐sense genomic RNA from the nucleocapsid, making the template accessible for translation. Ribosomal frameshifting drives the synthesis of viral replicase polyproteins (pp1a/pp1ab), which undergo autocatalytic cleavage by viral proteases to generate functional nonstructural proteins (nsps) that collectively orchestrate membrane remodeling [25]. The resulting replication–transcription complex (RTC) anchored to double‐membrane vesicles (DMVs) executes dual RNA synthesis: continuous negative‐strand replication and discontinuous transcription of nested subgenomic RNAs (sgRNAs) via leader–body fusion mechanisms. Concurrently, structural proteins (S, M, and E) traffic through ER–Golgi networks, converging with nucleocapsid‐encapsidated genomic RNA at ERGIC microdomains for virion assembly [26]. Progeny virions subsequently engage Golgi‐derived transport vesicles, leveraging Ca^2+^‐dependent SNARE protein interactions during exocytosis to achieve extracellular release—a spatiotemporally coordinated process regulated through nsp3‐mediated deubiquitinating and exoribonuclease proofreading activity of nsp14 that ensures replication fidelity (Figure 1) [27].

**Figure 2:**
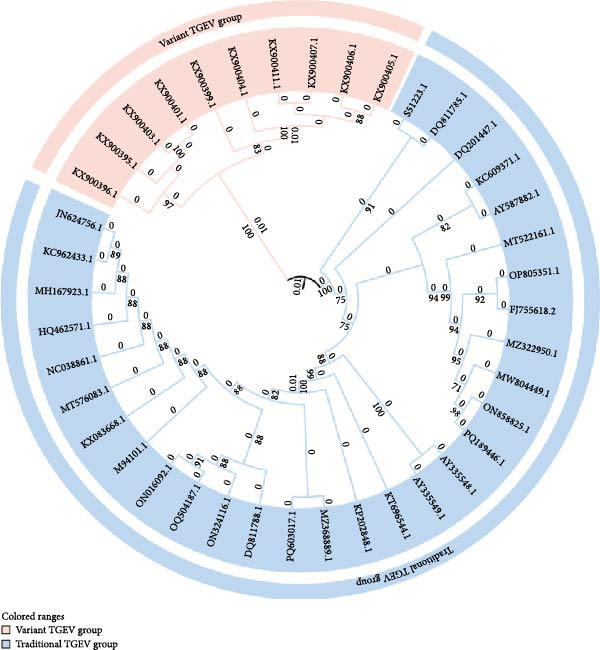
Phylogenetic analysis of TGEV strains based on the nucleotide sequences of the S gene [6]. A phylogenetic tree was constructed using the maximum likelihood method in MEGA 7.0 software based on 40 published TGEV S gene sequences, with bootstrap values calculated from 1000 replicates. The traditional strains are indicated in blue, while the variant strains are shown in pink.

**Figure 3:**
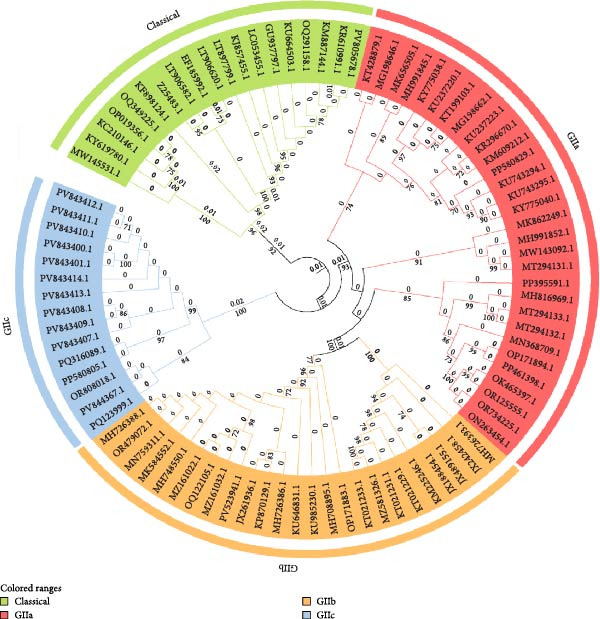
Phylogenetic analysis of PEDV genomes (excluding UTRs) [31]. A phylogenetic tree was constructed using the maximum likelihood method in MEGA 7.0 software based on 91 published PEDV S gene sequences, with bootstrap values calculated from 1000 replicates. The classical strains are green; the emerging GIIa, GIIb, and GIIc strains are red, orange and blue, respectively.

The error‐prone nature of RNA–dependent RNA polymerase (RdRp) serves as a fundamental driver of coronavirus genetic diversification, with phylogenetic reconstructions demonstrating clade‐specific mutations in the RBD of the S protein that confer selective advantages in host adaptation and immune evasion through structural modifications of viral surface epitopes [28]. Comparative genomic analyses revealed distinct evolutionary patterns between PEDV and TGEV. TGEV exhibits parallel evolutionary dynamics, reflected in the phylogenetic divergence between classical strains (Purdue and Miller) and variant strains (Figure 2) [29]. Notably, mutations clustered within residues 138−194 appear to mediate host spectrum expansion through conformational changes affecting sialic acid receptor binding specificity, suggesting a conserved mechanism for coronavirus host adaptation across genera [30]. PEDV evolution is characterized by the emergence of two principal genotypes: the classical variant (GI) and a global epidemic variant (GII). In recent epizootics, sublineages within the GII genotype, particularly, GIIa, GIIb, and GIIc, have become dominant. Their fitness has been linked to critical amino acid substitutions, such as those within positions 499–504 of the S1 subunit, which enhance enterocyte tropism through increased receptor binding affinity while simultaneously enabling evasion of neutralizing antibody recognition (Figure 3) [31–33]. Bayesian coalescent analyses of temporal phylogenies have identified interspecies recombination hotspots between animal and human CoVs, particularly at genomic regions encoding structural proteins [32, 34, 35]. These findings collectively highlight the role of RdRp‐driven mutations and genomic recombination in shaping the evolutionary trajectories of both zoonotic and veterinary CoVs, underscoring the need for integrated surveillance across species barriers.

### 2.2. Epidemiology, Transmission, and Host Dynamics of PEDV and TGEV

PEDV and TGEV, members of the Coronaviridae family, represent formidable threats to global swine production. PEDV, first isolated in Europe during the 1970s, has since undergone cosmopolitan dissemination, marked by cyclical outbreaks with notable intensity in Asian regions. Epidemiological records document peak mortality rates exceeding 80% among neonatal piglets during severe PEDV epidemics [2]. Since 2010, porcine epidemic diarrhea (PED) has emerged as a highly prevalent and devastating disease in China. In 2020, Tan et al. [36] conducted a comprehensive analysis summarizing the nationwide prevalence of PED in China since 2010. These findings revealed that PED cases were documented across nearly all provinces in China over this period, highlighting the extensive geographical spread of the disease. Conversely, TGEV, with a historical presence dating to the early 20th century, exhibits an endemic–epizootic pattern, with sporadic explosive outbreaks punctuating its otherwise stable circulation [11, 30]. TGEV was initially documented in the United States in 1946. Subsequently, it has been disseminated into 13 countries across Europe, Asia, Africa, and South America. In China, TGEV is predominantly distributed in nine provinces, spanning the eastern, northern, and central regions of the country. The transmission dynamics of these enteric CoVs converge predominantly via the fecal‒oral route, with contaminated feed matrices, potable water sources, and inanimate fomites serving as principal vectors. While shared transmission modalities exist, TGEV uniquely demonstrates aerosol‐mediated spread, a trait enabling rapid intrafarm and interfarm dissemination [37]. This aerobiological transmission mechanism underscores the heightened epidemic potential of TGEV in densely populated swine production systems.

Host susceptibility profiles exhibit striking ontogenic variation, with neonates constituting the most vulnerable cohort. PEDV–induced enteritis precipitates acute dehydration cascades, often culminating in mortality within 72 h postinfection [31]. In contrast, mature swine typically manifest subclinical infections or act as asymptomatic shedders, facilitating viral persistence within herds. Pathologically, both viruses target intestinal enterocytes, instigating villus atrophy and subsequent malabsorption syndrome [38]. Comparative genomics reveals a fundamental divergence in the host range: TGEV remains strictly adapted to Suidae, whereas PEDV exhibits broader tropism. This genetic plasticity endows PEDV with evolutionary flexibility reminiscent of zoonotic CoVs, portending potential cross‐species emergence events. Longitudinal genomic surveillance, informed by next‐generation sequencing technologies, is imperative for elucidating evolutionary trajectories and preempting antigenic shifts. Integration of phylodynamic analyses with epidemiological data will enable the development of predictive models to optimize biosecurity strategies and vaccine design, thus, safeguarding global pork production systems against these evolving viral threats [39].

## 3. Overview of the PEDV and TGEV Vaccines

Inactivated vaccines and live‐attenuated vaccines represent two major classes of traditional vaccines against porcine CoVs. Inactivated vaccines are produced through viral propagation followed by chemical inactivation (e.g., the use of formaldehyde or *β*‐propiolactone) [40], whereas live attenuated vaccines are generated via serial passaging in nonnatural hosts or cell cultures, a process that induces genomic mutations resulting in reduced virulence. Over the past few decades, these conventional vaccine platforms, particularly when formulated as polyvalent vaccines, have significantly contributed to the control of porcine coronavirus outbreaks. However, challenges persist, including incomplete cross‐strain protection, potential reversion to virulence of attenuated strains, and difficulties in eliciting mucosal immunity against enteric CoVs [41–43] (Table 1).

**Table 1:** Advances in vaccine research.

Immunogen	Design strategy	Models	Results	Advantages	Overall concerns	Reference
Inactivated vaccine						
Inactivated PEDV‐GS10	Inactivated PEDV‐GS10 strain	Pregnant sows	Effective immune response and protection in piglets	Safety, easy administration, scalability	Potential immune stress, lack of mucosal immunity, transient protection duration	[44]
TGEV + PEDV	Combined inactivated vaccine (JL strain + HN‐1 strain)	Piglets	Increased neutralizing antibody titers and protection rates for piglets	Bivalent, scalability	Potential immune stress, lack of mucosal immunity, transient protection duration	[45]
PEDV	Flagellin‐adjuvanted inactivated vaccine	Piglets	Enhanced immune protection against PEDV challenge	Strong immune response	Lack of mucosal immunity	[46]
TGEV	Nano silicon adjuvant‐enhanced inactivated vaccine	NHPs	Immunogenic and nearly sterile immunity	Humoral and cellular immune responses	Lack of mucosal immunity	[47]
Live attenuated vaccine						
PEDV COE protein	Recombinant *Lactobacillus casei* expressing dendritic cell‐targeting peptide fused with COE protein	Piglets	Effective immune response and protection against PEDV	Safe and effective oral delivery, strong immune response	Compromised neutralizing antibody epitopes	[48]
TGE‐PED bivalent attenuated vaccine	Attenuated vaccine administered via the Houhai acupoint	Piglets and pregnant sows	Achieved a passive immunity protection rate of 85.1%	Cost‐effectiveness, rapid immune activation, reduced vaccination dose	Difficulty in delivery	[49]
Recombinant chimeric TGEV‐PEDV	Recombinant chimeric virus	Piglets	Provides protection against virulent PEDV	Cross‐protection against TGEV and PEDV	Potential risk of reversion to virulence, antigenic mutation	[50]
Subunit vaccine						
PED COE domain subunit vaccine	Subunit vaccine based on COE domain replacement of flagellin domain D3	Mice	Improved specific humoral and mucosal immunity	Enhanced immune response	Lack of delivery carrier and mucosal immunity	[51]
S protein heptad repeat‐based nanoparticle vaccine	Nanoparticle vaccine targeting the dominant epitope of the S protein	Mice	Robust humoral and cellular immune responses	Strong immune response, potential for cross‐protection		[52]
Virus‐like particle vaccines						
PEDV‐VLP	VLPs formulated with CCL25/28 chemokines	Pigs	Induced systemic and mucosal immune protectivity	Strong immune response	Manufacturing challenges	[53]
VLPs with epitopes from PEDV and TGEV	VLPs with epitopes incorporated into self‐assembling ADDomer platform	Piglets	Provided clinical immune responses	Strong immune response, potential for cross‐protection		[54]
Genetically modified plant vaccines						
PEDV antigen fused to poly‐Fc of IgG	Expression in *Δ*XT/FT *Nicotiana benthamiana* plants	Piglets	Systemic and oral immunogenicity	Strong immune response, potential for mucosal protection	Low yield of antigen expression in plants	[55]
DNA vaccines						
S genes of TGEV and PEDV	Bivalent DNA vaccine delivered by attenuated *Salmonella* typhimurium	Mice or piglets	Effective immune response and protection against both TGEV and PEDV	Cross‐protection, efficient delivery system	Inefficient cellular uptake and nuclear entry compare to mRNA vaccines; genomic integration	[56]
mRNA vaccine						
PEDV S protein	mRNA vaccine encapsulated in LNPs (mRNA‐LNP)	Piglets	Humoral and cellular immune responses; active and passive protection	Strong immune response, potential for mucosal protection	Need of improvements in antigen engineering and delivery systems	[57]
Recombinant live vector vaccine						
PEDV S protein	Recombinant parapoxvirus expressing the S protein	Mice	Humoral and cellular immune responses	Effective immune response	Potential safety concerns, potential reversion to virulence, genetic instability leading to antigen loss during replication, and postvaccination adverse reactions	[58]
PEDV COE	Recombinant adenovirus vaccine encoding the COE	Mice	Elicited potent mucosal and systemic antibody responses	Strong immune response, potential for mucosal protection	—	[59]
PEDV S protein	Recombinant vesicular stomatitis virus expressing the S protein	Mice	Induced strong humoral immune response and neutralizing antibodies	Safe and effective platform	—	[60]
TGEV and PEDV antigens	Recombinant *Lactobacillus casei* expressing eGFP‐marked antigens	Mice	Demonstrated immunogenicity against both TGEV and PEDV	Safe and effective delivery system	—	[61]

Abbreviations: eGFP, enhanced green fluorescent protein; NHPs, nonhuman primates; PEDV, porcine epidemic diarrhea virus; S, spike; TGEV, transmissible gastroenteritis virus; VLPs, virus‐like particles.

### 3.1. Inactivated Vaccines

Inactivated PEDV vaccines are typically manufactured through viral propagation followed by inactivation via physical (e.g., heat treatment) or chemical (e.g., *β*‐propiolactone) methods [62]. These formulations may consist of whole virions or subunit antigens derived from fragmented viral components [63]. Notably, tissue‐derived inactivated vaccines based on epidemic virulent strains, such as the GS10 strain, have demonstrated robust immunogenicity in field trials. For example, Guo et al. [44] evaluated a GS10 strain–based vaccine administered intramuscularly in gravid sows and reported a 67% reduction in reproductive complications (miscarriage, preterm delivery, and fetal abnormalities) compared with unvaccinated controls. Passive immunity transfer was confirmed via enzyme‐linked immunosorbent assay (ELISA), with maternal antibodies in piglets maintaining protective titers (≥1:64) for 28 days postpartum, indicating durable neonatal protection [44].

To address the limited cross‐protection afforded by monovalent vaccines, recent efforts have focused on polyvalent formulations. Zang and Zhang [45] developed a bivalent inactivated vaccine targeting both PEDV (HN‐1 strain) and TGEV (JL strain) in neonatal piglets. By day 21 postvaccination, neutralizing antibody titers reached 1:512 (PEDV) and 1:256 (TGEV), correlating with clinical protection rates of 92% and 85%, respectively [45]. Commercial products predominantly include bivalent combinations (e.g., PEDV AJ1102/SCSZ‐1 strains with TGEV) and trivalent formulations incorporating PEDV CV777 strain, TGEV, and porcine rotavirus (PoRV). Nevertheless, antigenic interference between components remains a concern, with studies reporting up to a 40% reduction in PEDV‐specific antibody titers in trivalent formulations compared with their monovalent counterparts.

Contemporary adjuvant development has leveraged engineered NPs with optimized physicochemical properties, including high antigen adsorption capacity (> 500 mg/g), controlled release kinetics (<30% antigen leakage over 72 h), and thermostability (<10% efficacy loss after 30 days at 4–37°C). For instance, 50 nm NPs possessa fivefold greater surface area than traditional adjuvants, enhancing mucosal targeting efficiency (40% improvement in M‐cell uptake) and immunostimulation via Toll‐like receptor (TLR) pathway activation (threefold increase in dendritic cell maturation). The flagellin‐adjuvanted PEDV vaccine elevated secretory IgA (sIgA) levels by 2.3‐fold in piglets, achieving 95% protection against viral challenge [46], whereas the nanosilicon‐adjuvanted formulation induced fourfold higher IgG titers (vs. alum controls; *p* < 0.01) and 2.8‐fold increased IFN‐*γ* production in murine models [47].

Despite advantages in safety (adverse event rate <0.1%) and scalability (production cycle <30 days), inactivated vaccines exhibit limitations: suboptimal mucosal immunity (sIgA < 15 μg/mL in 60% of vaccinated sows), transient protection duration (<6 months in 45% of herds), and heterogeneous immune responses among gestating sows (20% seroconversion reduction in parity ≥5 sows). Critical knowledge gaps persist regarding nanosilicon adjuvant mechanisms, potentially involving TLR4/MyD88 pathway potentiation (30% increase in NF‐*κ*B activation) and cytokine storm modulation (65% reduction in IL‐6 overexpression). Future research should prioritize standardized efficacy metrics (per ISO 20776‐1) and multi‐omics integration (e.g., single‐cell RNA sequencing of gut‐associated lymphoid tissue [GALT]) to inform next‐generation mucosal vaccines and pan‐coronavirus strategies.

### 3.2. Live Attenuated Vaccines

Attenuated vaccines are biological preparations created by artificially weakening pathogens to retain immunogenicity while eliminating pathogenicity. Current strategies for developing attenuated vaccines predominantly utilize three approaches: naturally attenuated or cell culture‐adapted viral strains, live‐attenuated vaccines generated through serial passaging, and genetically engineered live vaccines developed via reverse genetics techniques. For example, Hou et al. [48] generated the PC177 attenuated strain by serially passaging the virulent PC21A strain in Vero cells, resulting in a 197‐amino acid deletion at the N‐terminus of the S protein. Oral immunization trials in piglets demonstrated effective immune protection. However, structural modifications of the S gene, while reducing virulence, may compromise neutralizing antibody epitopes, potentially diminishing their protective efficacy [48]. To address coinfections, attenuated PEDV strains are often combined with weakened TGEV and PoRV strains to create multivalent vaccines. This strategy leverages maternal immunity to provide synergistic protection against multiple enteric pathogens.

In practical applications, Tong [49] administered a TGE‐PED bivalent attenuated vaccine via Houhai acupoint injection to 3‐day‐old nursing piglets and pregnant sows, resulting in an 85.1% passive immunity protection rate. Compared with inactivated vaccines, this attenuated formulation reduces the immunization dose by 50% while offering cost‐effectiveness and rapid immune activation [49]. Pascual‐Iglesias et al. [50] innovatively developed a chimeric attenuated virus (rTGEV‐RS‐SPEDV) based on a TGEV backbone expressing the ectodomain of a U.S. PEDV virulent strain’s S protein. Challenge experiments in highly susceptible 5‐day‐old piglets revealed markedly reduced pathogenicity, with no weight loss or mortality observed. Histopathological analysis revealed minimal intestinal tissue damage compared with that caused by the wild‐type strains. Notably, this chimeric vaccine not only induced PEDV‐specific humoral immune responses with high‐titer neutralizing antibodies but also suppressed virulent strain colonization in the jejunum, as evidenced by undetectable fecal viral RNA levels, demonstrating robust cross‐protection.

Multivalent vaccine development has progressed significantly worldwide. China has commercialized trivalent inactivated vaccines and live attenuated vaccines based on the CV777 strain, with a GIIa genotype–targeted bivalent attenuated vaccine introduced in 2015. Japan predominantly employs the cell‐adapted 83P‐5 strain as a core component of attenuated live vaccines [64]. Immunologically, PEDV attenuated vaccines exhibit distinct advantages over their inactivated counterparts: their limited replication in vivo enables single‐dose immunization to elicit durable mucosal immunity, particularly sIgA production, which is critical for combating enteric viral infections. Additionally, attenuated vaccines minimize stress responses associated with multiple injections and reduce husbandry costs.

Nevertheless, challenges persist in attenuated vaccine applications. First, genetic compatibility between vaccine strains and circulating variants directly impacts protective efficacy, as ongoing viral evolution may facilitate immune escape. Second, safety concerns regarding potential virulence reversion and genetic recombination with wild‐type strains necessitate rigorous stability testing and molecular surveillance. Future vaccine development must balance immunogenicity with biosafety while enhancing the broad‐spectrum protective capacity of multivalent formulations. Continuous monitoring of field strain evolution and refinement of attenuation strategies will be essential to maintain vaccine effectiveness in dynamic epidemiological landscapes.

### 3.3. Subunit Vaccines

Subunit vaccines represent a class of biopharmaceuticals formulated with pathogen‐derived immunogens, such as structural proteins or polysaccharide conjugates, combined with adjuvants to enhance antigen‐specific immune responses. In PEDV vaccinology, the S glycoprotein has emerged as the principal antigenic target due to its dual role in mediating viral entry through host aminopeptidase N receptor binding and eliciting neutralizing antibodies. The S protein contains four critical neutralizing epitopes (COE, SS2, SS6, and 2C10) within its S1 domain, which collectively underpin its strong immunogenicity and utility in rational vaccine design. Pioneering studies have demonstrated the feasibility of integrating these antigenic domains with molecular adjuvants.

For example, Li et al. [51] engineered a chimeric COE‐flagellin fusion vaccine administered intramuscularly to 4‐week‐old piglets in a prime‐boost regimen, which elicited neutralizing antibody titers 3.2‐fold higher than those of monomeric COE vaccines. This immunogenicity translated to an 82% reduction in diarrheal incidence and preserved jejunal villus integrity (87.5 ± 3.2 μm vs. 53.1 ± 5.4 μm in controls), with mechanistic analyses revealing flagellin’s TLR5‐dependent activation of dendritic cells (2.5‐fold increase in CD80^+^ cells) and Th1‐polarized responses (IFN‐*γ*/IL‐4 ratio > 8:1) [51].

Recent advances in antigen delivery have further addressed traditional subunit vaccine limitations through nanostructured platforms. A notable innovation involves self‐assembling NPs constructed by fusing the heptad repeat (HR) domain of PEDV with *Helicobacter pylori* ferritin, which resulting in the formation of 24‐mer HR‐Fer NPs [52]. Murine immunization trials demonstrated that these NPs induced neutralizing antibody titers 4.7‐fold higher than those of soluble HR proteins (1:1280 vs. 1:272), achieving 95% protection against viral challenge. In vitro analyses revealed enhanced antigen uptake by bone marrow‐derived dendritic cells (73.5% ± 4.2% vs. 28.1% ± 3.8%) and upregulated major histocompatibility complex II (MHC‐II) expression (2.8‐fold increase), illustrating how particulate presentation amplifies cross‐presentation pathways. These findings align with the broader advantages of subunit vaccines over whole‐virion formulations, including enhanced safety (eliminating viral reactivation risks) and manufacturing scalability (> 2 mg/L yields in HEK293 systems). Furthermore, they concurrently activate humoral immunity (neutralizing IgG/IgA) and cellular responses (CD8^+^ T‐cell‐mediated viral clearance), achieving a 3‐log reduction in intestinal viral loads.

Despite these advancements, persistent challenges underscore the need for continued optimization. Approximately 38% of S protein–based vaccines fail to preserve native trimeric conformations, resulting in suboptimal neutralizing capacity [65]. Systemic immunization protocols often induce insufficient mucosal IgA (<15 μg/mL in gut lavage), a critical limitation given the enteric tropism of PEDV. Additionally, current expression systems struggle to maintain postfusion S protein stability beyond 8 weeks at 4°C, complicating field deployment. To bridge these gaps, future strategies should prioritize cryo‐EM‐guided stabilization of prefusion S conformations through targeted proline substitutions (e.g., S2‐PP mutation increases thermal stability by 12°C) and mucosal delivery platforms utilizing chitosan NPs (85% oral bioavailability) or M‐cell‐targeting ligands [66, 67]. Adjuvant innovation combining STING agonists (2.5‐fold increase in CD103^+^ dendritic cell recruitment) and IL‐17 potentiators (threefold sIgA enhancement) could synergistically enhance mucosal immunity, whereas longitudinal surveillance of emerging S protein variants (e.g., INDEL mutations at positions 56−59) could inform vaccine updates against heterologous strains [68, 69]. This integrated approach, which marries structural vaccinology with adaptive formulation design, is essential to translate the theoretical promise of subunit platforms into robust, field‐effective protection against evolving PEDV threats.

### 3.4. VLP Vaccines

VLPs are biomimetic nanostructures that replicate the structural and antigenic complexity of native virions, enabling them to provoke immune responses comparable to those of natural viral encounters while remaining noninfectious due to the absence of viral genetic material [70]. This intrinsic safety profile eliminates risks linked to pathogen replication, genetic recombination, or virulence restoration, positioning VLPs as superior alternatives to attenuated live or vector‐based vaccines [71–73]. Their capacity to display conformational epitopes in a repetitive spatial arrangement facilitates robust B‐cell activation and germinal center formation, driving potent humoral immunity. Current investigations into PEDV‐specific VLP vaccines predominantly utilize murine models for immunogenicity assessment. However, such models suffer from translational limitations, as mice lack susceptibility to PEDV infection and fail to recapitulate the pathophysiological manifestations observed in swine [74]. Consequently, neutralizing antibody titers derived from murine studies may not reliably predict protective efficacy in pigs, necessitating validation in target species under clinically relevant conditions, including viral challenge trials and field‐based evaluations of mucosal immunity.

PEDV and TGEV VLP vaccines are engineered through the co‐expression of viral structural proteins, such as the S, M, and E proteins, which self‐assemble into nonreplicative particles that retain antigenic authenticity. Hsu et al. [53] demonstrated this principle by coexpressing PEDV S, M, and E proteins in a baculovirus system, yielding VLPs capable of inducing PEDV–specific immunoglobulins and cellular immune responses in pigs following intramuscular administration. Importantly, the incorporation of the chemokine adjuvant CCL25/28, a combination known to promote lymphocyte trafficking to mucosal sites, synergistically enhanced systemic anti‐S IgG production and stimulated the secretion of mucosal IgA, a critical mediator of intestinal immunity. This dual‐modality approach not only amplified immune breadth but also conferred substantial protection in weaned piglets, as evidenced by reduced fecal viral shedding and preservation of the intestinal villus architecture. These findings underscore the pivotal role of adjuvant selection in tailoring immune responses to enteric pathogens, where mucosal immunity is indispensable for neutralizing infection at primary sites of viral entry [53].

Epitope‐focused VLP design leverages structural vaccinology to embed immunodominant epitopes within synthetic NP scaffolds. A seminal study integrated neutralizing epitopes from the PEDV S protein (SS2 and 2C10 regions) and TGEV S protein (A and D domains) into the ADDomer platform, a highly ordered adenovirus‐derived NP. Recombinant proteins (AD, AD‐p, AD‐t, and AD‐pt) produced via baculovirus expression were emulsified with the ISA 201VG adjuvant, generating multivalent VLP vaccines. The immunization of 4‐week‐old piglets elicited cross‐reactive neutralizing antibodies against both viruses, accompanied by heightened Th1/Th2 cytokine profiles (IFN‐*γ*, IL‐2, and IL‐4) and increased cytotoxic T lymphocyte (CTL) activity, indicative of robust cellular immunity. However, the immunogenicity of these epitope‐engineered VLPs remains inferior to that of conventional live or inactivated vaccines, which is likely attributable to suboptimal antigen density or conformational instability of engineered epitopes. To address this, advanced protein engineering strategies, such as the incorporation of disulfide bonds or trimerization motifs, could stabilize quaternary epitope conformations, thereby enhancing immune recognition and the magnitude of the response [54].

Manufacturing challenges in VLP production are multifaceted, particularly in downstream processing. Low yields during purification, often exacerbated by particle aggregation and host cell contaminant persistence, hinder scalability [75].Traditional methods such as density gradient ultracentrifugation are inefficient for industrial‐scale applications, while chromatographic approaches require extensive optimization due to the heterogeneous surface charge and hydrophobicity across VLP constructs [76]. Furthermore, the absence of rapid and high‐resolution analytical tools for assessing antigenic integrity complicates quality assurance. Emerging innovations, such as continuous bioprocessing systems and affinity tag‐based purification (e.g., His‐tag or Strep‐tag), promise to increase yield and reproducibility. Concurrently, advanced characterization techniques like cryo‐electron microscopy (cryo‐EM) and machine learning–driven epitope stability modeling enable atomic‐level validation of antigenic structure, accelerating process development and regulatory compliance.

Future directions in VLP vaccinology emphasize the convergence of structural precision, adjuvant innovation, and mucosal delivery. Cryo‐EM–guided epitope mapping and computational design of stabilized S protein trimers could refine antigenic fidelity, ensuring preservation of critical neutralizing epitopes [77]. Adjuvants targeting pattern recognition receptors (e.g., TLR3 agonists for dsRNA mimicry or STING agonists for cytosolic DNA sensing) may potentiate innate immune activation in GALT, aligning immune responses with the enteric pathogenesis of PEDV [68, 78, 79]. Oral or intranasal delivery systems employing enteric‐coated NPs or mucoadhesive polymers could further enhance mucosal immunization efficacy [80]. While epitope‐based VLPs present a safer paradigm, bridging the efficacy gap with traditional vaccines demands iterative design cycles, incorporating heterologous prime‐boost regimens and cross‐protective epitope selection. Parallel advancements in modular manufacturing platforms, such as cell‐free synthesis or plant‐based expression systems, will be critical to overcoming production bottlenecks, ensuring scalable, cost‐effective VLP vaccine deployment [81, 82]. Ultimately, the transition from preclinical promise to clinical utility hinges on rigorous challenge trials in swine, validating both immunogenicity and field‐level protection against evolving PEDV strains.

### 3.5. Genetically Modified (GM) Plant Vaccines

GM plant vaccines represent an innovative biotechnological approach in which immunoprotective antigen genes of PEDV are engineered into plant genomes for in planta expression. This strategy leverages plants as bioreactors to produce antigenic proteins capable of eliciting protective immune responses. To date, PEDV–derived antigens, such as the S protein and its immunodominant COE domain, have been successfully expressed in diverse plant species, including tobacco (*Nicotiana tabacum*), rice (*Oryza sativa*), and lettuce (*Lactuca sativa*), via transgenic or transient expression systems [83–85]. For example, oral administration of transgenic tobacco expressing the COE domain induced concurrent systemic and mucosal immune responses in mice, as evidenced by elevated serum IgG and mucosal IgA titers, confirming the immunogenic potential of plant‐based vaccines [55]. Similarly, Yin et al. [8] demonstrated the feasibility of expressing the PEDV S1 protein in tomatoes (*Solanum lycopersicum*), which, when fed to piglets, stimulated robust active immunity and conferred significant protection against PEDV challenge. These studies underscore the capacity of GM plants to function as dual‐purpose antigen production platforms and oral delivery vehicles.

Compared with traditional inactivated vaccines, GM plant vaccines offer distinct immunological and practical benefits. They inherently stimulate both systemic and mucosal immunity, which is critical for combating enteric pathogens such as PEDV, which primarily invade through the gastrointestinal mucosa. The plant cell wall acts as a natural adjuvant, protecting antigens from premature degradation and facilitating their gradual release in the gut, thereby enhancing antigen uptake by GALT. This dual immune activation, coupled with the cost‐effectiveness and scalability of plant‐based production, positions GM vaccines as promising alternatives for mass vaccination in livestock industries.

Despite their potential, GM plant vaccines face significant technical hurdles. A primary limitation is the low yield of recombinant antigen expression in plants, which is often insufficient to induce potent immune responses without repeated dosing. Furthermore, oral delivery poses bioavailability challenges: gastric acidity and proteolytic enzymes degrade a substantial portion of antigens before they reach intestinal immune inductive sites, drastically reducing effective antigen exposure. For example, while transgenic tomatoes elicited protective immunity in piglets, the required antigen dose was substantially higher than that of injectable vaccines, reflecting inefficient oral bioavailability. These constraints highlight the need for optimization of expression systems to boost antigen production and the development of encapsulation strategies to shield antigens from gastrointestinal degradation.

### 3.6. DNA Vaccines

DNA vaccines, also known as nucleic acid or genetic vaccines, function by introducing recombinant eukaryotic expression vectors encoding specific protein antigens directly into host organisms [86]. These vectors enable the in vivo expression of foreign genes, producing antigens that stimulate both humoral and cellular immune responses. This approach circumvents the need for pathogen cultivation or protein purification, offering a flexible and scalable platform for antigen presentation.

In a study by Zhang et al. [56], a bivalent DNA vaccine containing the S genes of TGEV and PEDV was delivered orally to 20‐day‐old piglets using attenuated *Salmonella Typhimurium* SL7207 as a bacterial vector. Following a prime‐boost regimen with two doses administered 14 days apart, the vaccine elicited concurrent serum IgG and mucosal IgA responses against both pathogens, demonstrating its capacity to induce dual‐pathogen immunity. The attenuated *Salmonella* strain serves as a targeted delivery system, colonizing the GALT after oral ingestion by infecting colonic mucosal cells and persisting within immune cells such as macrophages. This localized replication promotes sustained antigen exposure, resulting in robust mucosal immunity, which is essential for combating intestinal pathogens and producing systemic antibodies. Importantly, no adverse effects or mortality were observed at a dose of 10^11^ colony‐forming units (CFUs), underscoring the safety of this delivery platform. This study highlighted the unique advantage of oral DNA vaccines in synchronizing mucosal and systemic immune activation, as they outperform parenteral routes in eliciting gut‐specific antibody responses [56].

Despite the above advantages, DNA vaccines face inherent challenges. While they offer rapid design flexibility, thermostability, and cost‐effective scalability which are particularly advantageous for emerging infectious diseases, their immunogenicity often lags behind that of mRNA vaccines because of inefficient cellular uptake and nuclear entry of plasmid DNA. Theoretical concerns regarding genomic integration, although rare in practice, necessitate rigorous long‐term safety monitoring [87, 88]. Current strategies to enhance efficacy include codon optimization, electroporation‐assisted delivery, and the coexpression of molecular adjuvants with target antigens.

In the future, advancements in vector engineering and delivery systems will be critical to overcoming existing limitations. For example, integrating CRISPR‐Cas9‐guided mechanisms to avoid genomic insertion could mitigate safety concerns. Hybrid platforms combining DNA vaccines with mRNA or protein boosts may leverage synergistic immunogenicity, whereas oral *Salmonella*‐vectored systems hold promise for mass immunization in livestock because of their ease of administration and dual‐action protection against cocirculating pathogens like TGEV and PEDV. As DNA vaccine technology converges with innovations in synthetic biology and mucosal immunology, its role in pandemic preparedness and veterinary health is poised to expand, provided that challenges in immunogenicity and delivery efficiency are systematically addressed through iterative design and preclinical validation.

### 3.7. mRNA Vaccines

mRNA vaccines function by delivering synthetic mRNAs encoding target antigens into host cells via specialized delivery systems, such as lipid NPs (LNPs). Upon cellular uptake, mRNAs are translated into antigenic proteins, which are processed and presented to the immune system, where they elicit both humoral and cellular immune responses [89]. This platform bypasses the need for live pathogens or protein purification, enabling rapid and scalable vaccine development. In a study by Zhao et al. [57], two LNP‐encapsulated mRNA vaccine candidates were developed: one encoding the full‐length S protein of PEDV and another encoding a multiepitope chimeric S protein (Sm). Subcutaneous immunization of 6‐week‐old BALB/c mice with these constructs followed by a 14‐day booster revealed that the full‐length S mRNA vaccine induced significantly stronger antibody titers and T‐cell responses than the Sm variant did, highlighting the importance of structural integrity in antigen design for optimal immunogenicity.

The translational potential of these vaccines was further evaluated in swine models through active and passive immunization strategies. For active immunity, 1‐day‐old piglets received subcutaneous S mRNA‐LNP vaccinations in the neck region, were boosted on Day 14, and were challenged with PEDV on Day 28. In passive immunity trials, pregnant sows were immunized with the same vaccine, and their 5‐day‐old piglets were orally challenged with PEDV. Both approaches demonstrated robust PEDV‐specific immune responses in piglets, with active immunization conferring direct protection and passive immunization transferring immunity via colostral antibodies. These findings underscore the dual utility of mRNA vaccines in neonatal and maternal vaccination programs, which are critical for controlling PEDV outbreaks in swine populations. However, a major limitation emerged from the genetic diversity of PEDV, which is classified into four subtypes (GIa, GIb, GIIa, and GIIb). Current mRNA vaccines exhibit limited cross‐neutralizing activity across these subtypes, likely owing to antigenic drift in the S protein, a phenomenon that undermines the efficacy of existing PEDV vaccines. This underscores the need for next‐generation mRNA vaccines engineered to target conserved epitopes or incorporate multivalent designs to broaden cross‐protective coverage [57].

Despite demonstrating distinct advantages such as rapid development timelines, high safety profiles (no risk of infection), strong adjuvant‐free immunogenicity, and cost‐effective scalable manufacturing, which are particularly valuable for combating emerging or rapidly evolving pathogens, mRNA vaccines still face persistent technical challenges [90–92]. The inherent instability of mRNA necessitates ultracold storage and transportation, complicating distribution in resource‐limited settings. Furthermore, transient immune responses may require frequent booster doses, while potential allergic reactions to LNP components and unresolved long‐term safety concerns warrant further investigation [93]. A critical barrier lies in the delivery process: extracellular ribonucleases in blood and tissues rapidly degrade unprotected mRNA, necessitating advanced encapsulation technologies to ensure cellular delivery. Current LNP systems, while effective, may introduce cytotoxicity or interfere with immune signaling due to carrier material interactions, emphasizing the need for safer and biodegradable delivery platforms.

Future advancements in mRNA vaccine design must address these limitations through innovations in both antigen engineering and delivery systems. For cross‐subtype protection, computational prediction of conserved S protein epitopes or fusion of multiple subtype‐specific antigens into a single mRNA construct could enhance breadth [94]. Stabilization of mRNAs via nucleotide modification or novel lipid formulations may improve thermostability, easing logistical constraints [95]. Concurrently, developing ribonuclease (RNase)‐resistant delivery vehicles or alternative routes of administration (e.g., intranasal) could enhance mucosal immunity and reduce the degree of degradation risk. Integrating immunomodulatory sequences into mRNA constructs may further prolong immune memory, reducing the booster frequency [96, 97]. As these refinements progress, mRNA technology holds promise not only for veterinary applications against PEDV but also as a versatile platform for addressing global infectious disease challenges, provided that safety, stability, and cross‐protection hurdles are systematically overcome through iterative preclinical and clinical optimization [98].

### 3.8. Recombinant Live Vector Vaccines

Recombinant live vector vaccines are engineered by integrating foreign antigen‐encoding genes into the genome of attenuated viral or bacterial vectors, enabling in vivo expression of target antigens to elicit immune responses. These vaccines are broadly categorized into viral or bacterial vector‐based platforms, each leveraging distinct biological mechanisms for antigen delivery. For instance, Hain et al. [58] constructed a recombinant Orf virus (ORFV) expressing the PEDV S protein, which elicited robust IgG and IgA responses in piglets following intramuscular immunization, alongside Th1/Th2‐balanced cellular immunity. Yan et al. [59] further explored mucosal delivery via a human adenovirus (Ad5) vector expressing the PEDV COE epitope. Intranasal administration in mice outperformed intramuscular injection inducing mucosal IgA, underscoring the importance of the delivery route in optimizing immunity [59]. Another innovative approach by Ke et al. [60] utilized a truncated vesicular stomatitis virus (VSV) vector to incorporate the PEDV S protein into viral particles. The immunization of pregnant sows conferred passive immunity to piglets via colostral transfer of neutralizing antibodies, effectively protecting neonates against lethal PEDV challenge [60].

In addition to monovalent designs, multivalent vaccine strategies have advanced through the engineering of a recombinant PEDV‐M bivalent vaccine (rPEDV‐PoRV‐VP7), in which the PoRV VP7 gene replaces the ORF3 region of an attenuated PEDV strain [9]. This chimeric virus retained replication kinetics comparable to those of wild‐type PEDV in vitro and stably expressed VP7 in infected cells. Vaccination of piglets induced dual‐specific IgG/IgA antibodies and neutralizing activity against both pathogens without adverse effects, although cross‐protection against other enteric viruses and the durability of immunity remain to be elucidated.

Parallel advancements have been made in bacterial vector systems, which exploit distinct biological pathways compared to viral platforms. Attenuated *Salmonella*, *Bacillus subtilis*, and *Lactobacillus strains* engineered to express PEDV S protein domains consistently induce IgG/IgA responses in preclinical models, although field validation of their protective efficacy awaits confirmation [99, 100]. The intestinal tropism of *Salmonella* vectors exemplifies their mechanistic advantage, as these organisms traverse intestinal epithelia to deliver antigens directly to GALT. This natural targeting facilitates dendritic cell activation, T‐cell priming, and B‐cell differentiation in Peyer’s patches, supported by inherent adjuvant effects from pathogen‐associated molecular patterns such as LPS. This dual functionality enables durable immune memory through plasma cell generation and mucosal protection.

Technological diversification is illustrated by *Lactobacillus lactis* expressing the PEDV COE antigen and *Lactobacillus* casei‐based bivalent vaccines targeting PEDV and TGEV, both demonstrating antigen‐specific serum IgG and mucosal IgA induction in animal models without adjuvants [61]. These probiotic‐based systems highlight the potential for safe mucosal immunization through oral delivery.

However, practical challenges persist. Vector‐related risks include potential reversion to virulence, genetic instability leading to antigen loss during replication, and postvaccination adverse reactions [101]. Furthermore, the immunological focus on vector components may dilute responses to target antigens. Addressing these limitations necessitates rigorous vector attenuation, stability testing across passages, and balanced antigen–vector immunogenicity profiles. Future optimization may involve synthetic biology tools to enhance antigen loading, stabilize genetic constructs, and refine delivery systems for targeted mucosal or systemic immunity, ultimately advancing these platforms toward robust, multispecies vaccine solutions (Table 2).

**Table 2:** Comparison of the main vaccine platform characteristics of PEDV and TGEV.

Vaccine platform	Principle of immunization	Main advantages	Key limitations	Research and development maturity
Inactivated vaccines	Processed and presented by antigen‐presenting cells, mainly inducing humoral immunity (IgG)	High safety, mature production process, stable	Weak mucosal immunity, short‐protection duration, and limited cross‐protection against heterologous strains	Commercialization and wide use
Live attenuated vaccines	Simulate natural infection and induce humoral (IgG, IgA), cellular (CTLs), and mucosal immunity	Comprehensive immune response (humoral, cellular, mucosal), strong protection, and low cost	Risk of toxic rebound	Commercialization and wide use
Subunit vaccines	Express key antigenic proteins in the virus (e.g., S protein or its COE domain) and combine them with adjuvants to enhance the immune response	High safety, clear composition, and easy production scalability	Weak immunogenicity, often needing adjuvants, lacking strong mucosal immunity and insufficiently inducing cellular immunity	Most in development, some in clinical trials
Virus‐like particle vaccines	Hollow particles containing one or more structural proteins of a virus	Natural viral conformation, strong immunogenicity, high safety	Complex production, low yield, high cost, and challenges in conformational stability	Preclinical studies
Genetically modified plant vaccines	Introduce vaccine‐related genes into plants and administer orally to stimulate the intestinal mucosal immune system	Low cost, easy to scale production, oral induction of mucosal immunity	Low antigen expression, easy degradation in digestive tract during oral delivery, and unstable immunogenicity	Early development stage
DNA vaccines	Recombinant eukaryotic expression vector codes for protein antigen. In vivo antigen expression activates immunity and induces humoral and cellular immune responses	Rapid development, simple production, good thermal stability, induce comprehensive immunity	Low transfection efficiency, weak immunogenicity, and potential genomic integration risk	Most in preclinical studies, some veterinary vaccines approved
mRNA vaccines	The mRNA delivering the coding antigen is produced to generate the antigen and trigger a comprehensive immune response	Rapid development, efficient induction of comprehensive immunity, no infection risk, and easy to update for variants	Exhibits poor stability and demands refrigeration. The LNP delivery system might induce adverse effects, with restricted cross‐protection against heterologous strains	Rapid development, some in clinical trials, mature technology platforms
Recombinant live vector vaccines	Use attenuated virus/bacteria vectors to deliver antigen genes	Comprehensive immunity (especially mucosal immunity), safety	Vector preexisting antibody interference, genetic recombination risk	Some veterinary vaccines approved, new vectors under development

In summary, several studies cited in this review utilized mouse models to evaluate immunogenicity; however, this model has inherent limitations when applied to porcine pathogens. Since PEDV and TGEV, among other porcine pathogens, do not naturally host in mice, the susceptibility of mice is low, and they lack the corresponding specific intestinal receptors, making it impossible to reproduce the typical enteric epithelial lesions and viral replication cycles observed in pigs [102]. Furthermore, fundamental differences between the immune system structure, gut microbiota composition, and mucosal immune responses of mice and pigs mean that neutralizing antibody titers or cellular immune responses observed in mouse models may not accurately reflect protective efficacy in the target species [103, 104]. Therefore, although mouse models are indispensable for high‐throughput preliminary screening of vaccine candidates, the immunological data obtained must be interpreted in the context of the specific target species. Subsequent studies should employ porcine models for rigorous viral challenge experiments to validate the mucosal immune responses elicited by the vaccine and their overall protective efficacy.

## 4. Landscapes and Key Aspects of the PEDV and TGEV Vaccines

As reviewed, currently available vaccines have demonstrated partial efficacy against emerging pathogenic intestinal coronavirus in swine, but exhibit several limitations. These vaccine candidates fail to provide adequate on‐site protection at the intestinal mucosa. Additionally, protection against homologous and heterologous strains of PEDV and TGEV remains inadequate, as current vaccines lack the ability to induce broad cross‐protective immunity. Furthermore, comprehensive risk mitigation strategies must be systematically implemented to address genomic instability‐induced virulence reversion events, particularly in live attenuated vaccine development. These shortcomings have become increasingly apparent with the continuous emergence of novel highly pathogenic PEDV and TGEV variants, highlighting the urgent need for next‐generation vaccine platforms that can overcome the immunological and practical limitations of traditional approaches. To objectively evaluate the efficacy of these novel candidates and enable direct comparison across platforms, the field calls for the establishment of a unified framework of standardized metrics.

### 4.1. Mucosal Immunity and Different Delivery Routes

**Figure 4:**
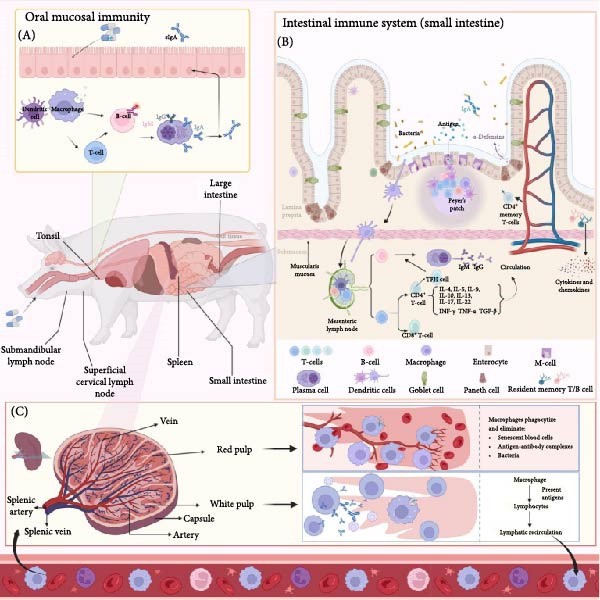
Potential entry points for mucosal immunity against porcine enteric coronavirus. (A) Oral‐associated lymphoid tissues (such as tonsils and cervical lymph nodes) recognize pathogenic antigens and initiate mucosal immune responses, transmitting immune signals to the entire body through the lymphatic circulation. (B) GALTs (including Peyer’s patches and mesenteric lymph nodes) mediate antigen presentation and immune cell activation locally in the intestine, forming a specific mucosal barrier. (C) Systemic immunity integrates mucosal and systemic immune responses through the bloodstream, disseminating protective antibodies and memory cells to distant tissues.

Mucosal immunity refers to the immune response elicited by mucosal‐associated lymphoid tissues (MALTs) located in the gastrointestinal tract, respiratory tract, urogenital tract, and exocrine glands against invading pathogens or vaccine antigens. Ideal mucosal vaccines exhibit unique mucosal tropism and inherent adjuvant properties. By mimicking natural infection routes, these vaccines establish a multitiered defense system at mucosal portals of pathogen entry (Figure 4). This strategy achieves spatiotemporal precision in immune regulation through two synergistic mechanisms: (1) induction of sIgA to form a molecular barrier on mucosal surfaces and (2) activation of mucosal tissue‐resident memory T (TRM) cells. This dual activation is particularly advantageous against highly mutable respiratory viruses. Preclinical studies have confirmed that mucosal CD8^+^ T‐cells targeting conserved viral epitopes exhibit durable and broad‐spectrum cross‐protective effects, surpassing transient neutralization by antibodies prone to viral escape [105–116].

The induction of mucosal immunity in the gastrointestinal tract represents a critical objective in vaccine development against PEDV and TGEV, as both pathogens predominantly target the small intestinal epithelium, causing severe enteric damage and systemic dehydration [59]. In addition to the classical fecal–oral transmission route, emerging evidence highlights the airborne dissemination of PEDV via compromised pulmonary endothelial barriers, expanding our understanding of transmission dynamics and emphasizing the need for multisite mucosal protection [117]. Central to mucosal defense mechanisms are sIgA‐mediated neutralization at epithelial surfaces, which prevents viral attachment and subsequent cellular entry through steric hindrance and immune exclusion [118]. This front line immunological barrier not only reduces the viral load prior to systemic invasion but also mitigates interindividual transmission, making mucosal vaccination a strategic intervention for controlling enteric coronavirus outbreaks in swine populations. Intranasal immunization has unique immunological merits, requiring lower antigen doses while simultaneously engaging both respiratory and intestinal mucosal networks. Preclinical studies of SARS‐CoV‐2 vaccines delivered via nasal sprays or nebulizers in nonhuman primates revealed enhanced mucosal IgA responses compared to parenteral routes, suggesting translational potential for optimizing PEDV and TGEV vaccine formulations [119]. The anatomical connectivity between nasal‐associated lymphoid tissue (NALT) and GALT facilitates dual‐site immune activation, creating a coordinated defense system against pathogens via complex transmission routes.

From an immunization strategy perspective, mucosal delivery offers dual breakthroughs: stealth properties: preventing the antivector immunity encountered with injectable vaccines, enabling sequential prime‐boost regimens and enhancing immunogenicity [103]. User‐friendly administration: Noninvasive routes (e.g., oral and inhaled) not only improve vaccine accessibility (e.g., oral bait vaccines for wildlife immunization) but also precisely interrupt respiratory/enteric transmission chains. Critically, this “bionic” immunization activates the mucosal‐systemic immune axis, coupling local mucosal immunity with systemic protection via the common mucosal immune system, thereby innovating herd immunity strategies [120]. Early establishment of immune defenses at infection portals reduces viral loads by > 90%, while redosable administration supports sustainable immunity against rapidly evolving pathogens [121].

### 4.2. Novel Nucleic Acid Vaccines

The accelerated development and global implementation of mRNA vaccines during the COVID‐19 pandemic have revolutionized vaccinology [122], yet persistent challenges, including thermodynamic instability requiring ultracold storage and transient antigen expression, underscore the need for advanced nucleic acid platforms. Next‐generation solutions such as self‐amplifying mRNA (saRNA) and circular RNA (circRNA) vaccines are emerging as paradigm‐shifting technologies, offering enhanced stability, prolonged immunogenicity, and translational flexibility to address these limitations [123].

#### 4.2.1. saRNA Vaccines

**Figure 5:**
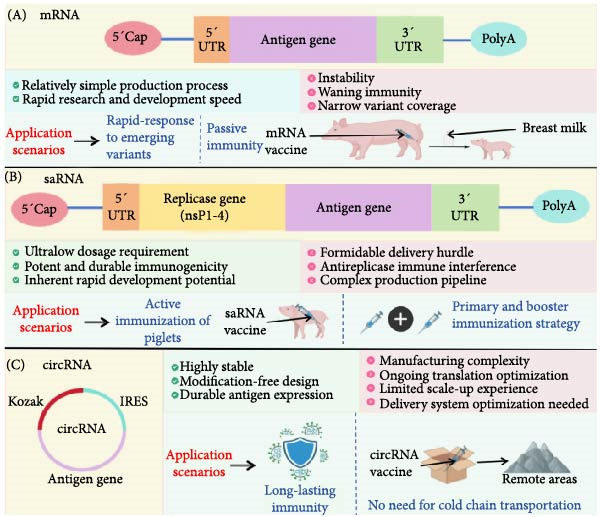
Comparative features of mRNA, saRNA, and circRNA vaccine platforms. (A) mRNA vaccine platform highlights features such as the 5^′^ cap, antigen gene limitations, and rapid response to emerging variants. (B) saRNA vaccine platform emphasizes replicase genes, ultralow dosage, durable immunogenicity, and challenges in delivery and manufacturing. (C) circRNA vaccine platform focuses on high stability, durable antigen expression, and suitability for long‐lasting immunity without cold chain requirements.

saRNA vaccines utilize alphavirus‐derived RNA replicase to enable intracellular RNA amplification, achieving sustained antigen production and potent immune activation at ultralow doses (1/10–1/100th of conventional mRNA requirements) [124]. This platform synergizes manufacturing scalability driven by reduced RNA quantities per dose, with improved safety profiles via minimized reactogenicity from lower nucleic acid loads [125]. Its capacity for multivalent formulations allows concurrent targeting of cocirculating pathogens, a critical advantage in combating viral evolution. Preclinical studies highlight the broad‐spectrum efficacy of saRNAs against rabies, Ebola, and porcine CoVs (PEDV and TGEV), where prolonged antigen expression enhances cross‐protective immunity and reduces booster frequency, which is a pivotal feature for cost‐effective veterinary mass immunization [126]. Clinical trials have confirmed durable neutralizing antibody induction and T‐cell memory formation, with therapeutic extensions into oncology through tumor‐specific antigen delivery. This dual prophylactic‐therapeutic versatility positions saRNAs as cornerstone technologies for pandemic preparedness, precision immunotherapy, and large‐scale agricultural applications (Figure 5).

#### 4.2.2. circRNA Vaccines

circRNA vaccines employ covalently closed‐loop structures devoid of free 3^′^/5^′^ termini, conferring unparalleled resistance to exonuclease degradation [127]. This structural robustness enables extended antigen expression (> 21 days in murine models) and reduces cold‐chain dependency—a transformative feature for resource‐limited settings. Thermostable formulations retain immunogenicity after several weeks at 4°C, overcoming a major logistical barrier in veterinary and global health. Compared with linear mRNAs, preclinical models of circRNA vaccines encoding the rabies glycoprotein or SARS‐CoV‐2S protein demonstrate superior antibody titers and germinal center activation, which is attributable to persistent antigen presentation. Notably, the compatibility of circRNAs with mucosal delivery enhances their utility against enteric pathogens such as PEDV and TGEV, where intestinal IgA responses are pivotal for protection [128]. Emerging oncology applications further exemplify its adaptability, with circRNAs encoding tumor neoantigens eliciting robust cytotoxic T‐cell responses in preclinical cancer models [129] (Figure 5).

#### 4.2.3. Synergistic Potential and Future Directions

The complementary strengths of saRNAs (dose efficiency and prolonged immunogenicity) and circRNAs (thermostability and mucosal adaptability) redefine nucleic acid vaccines as dual‐purpose tools for human and veterinary medicine. For porcine CoVs, these platforms address historical gaps in cross‐protection, strain adaptability, and field‐compatible storage. Future research must prioritize delivery optimization (e.g., LNP engineering for intestinal mucosa targeting) and comprehensive safety evaluations in swine models. By merging the rapid design flexibility of nucleic acid platforms with advanced formulation science, next‐generation vaccines could simultaneously achieve pandemic resilience and cost‐effective livestock protection, ultimately alleviating the global economic toll of PEDV, TGEV, and emerging zoonotic threats.

### 4.3. AI–Assisted Vaccine Design

**Figure 6:**
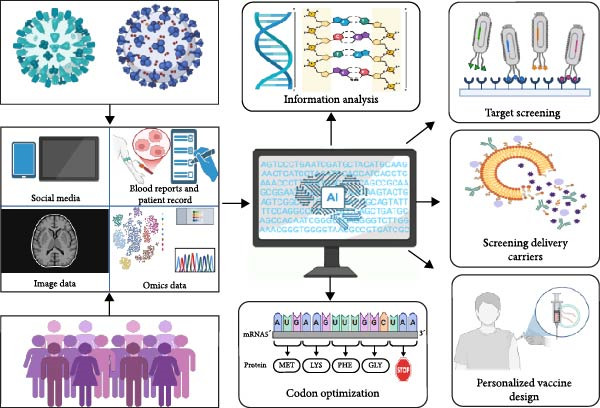
Artificial intelligence–assisted vaccine design. This schematic outlines the AI–driven vaccine design pipeline, which integrates multisource data analysis (e.g., blood metrics, clinical records, omics, and social media) to identify high‐potential immunogenic targets. Machine learning and molecular modeling optimize delivery vectors (e.g., LNPs) for specificity and biocompatibility, whereas AI–guided codon optimization enhances antigen expression and folding in host cells. Finally, individual genetic and immunological profiles inform personalized vaccine models, enabling end–to‐end automation from target discovery to sequence engineering for efficient and precision vaccine development.

The integration of AI into vaccine design holds transformative potential for combating porcine CoVs. Leveraging computational power to analyze complex biological datasets, including viral protein structures, host–pathogen interaction networks, and genomic sequence‐AI–driven platforms, can significantly accelerate antigen discovery and immunogen optimization, streamlining a traditionally labor‐intensive process while reducing the time and financial expenditures associated with conventional empirical approaches [130, 131]. Advanced machine learning algorithms enable high‐throughput prediction of antigenic epitopes and immunogenicity profiles, allowing researchers to prioritize candidate antigens with precision, thereby minimizing experimental redundancy and accelerating preclinical validation [132] (Figure 6).

Generative AI models and molecular dynamics simulations further enhance vaccine design by predicting structural modifications to improve immunogen stability and broaden epitope coverage, which is critical for addressing viral heterogeneity and emerging variants. Beyond antigen design, AI facilitates the discovery of novel adjuvants through virtual screening of compound libraries, identifying molecules with optimal immunomodulatory properties and safety profiles to increase vaccine efficacy. These computational strategies are complemented by AI’s capacity to optimize vaccination protocols, including dose regimens and administration schedules, through predictive modeling of preclinical and clinical data. Such models balance immune response durability against reactogenicity risks, ensuring robust mucosal and systemic immunity while minimizing adverse effects—a critical consideration for livestock vaccination programs requiring mass deployment.

The convergence of AI with multiomics data (e.g., transcriptomics and proteomics) and advanced delivery systems (e.g., NP carriers and mucosal adjuvants) promises to redefine vaccine development pipelines. By enabling rapid iteration of vaccine candidates tailored to evolving viral strains and host immune landscapes, AI–driven platforms not only enhance the precision and efficiency of TGEV/PEDV vaccine design but also establish a scalable framework for addressing future zoonotic threats. This paradigm shifts toward data‐driven vaccinology, underscoring AI’s pivotal role in achieving cost‐effective, broadly protective solutions for global swine health and food security.

### 4.4. Piglet Passive Immunity

PEDV infection in neonatal piglets leads to substantial mortality rates (20%–50%) and growth retardation, primarily attributed to the underdeveloped immune systems of young swine. This clinical manifestation has resulted in significant economic losses in global pork production systems. A notable example occurred during the 2013−2014 PEDV outbreak in the United States, which resulted in a 3.2% annual reduction in swine populations [133]. Despite extensive research efforts, current commercial vaccines are insufficient for providing robust protection to newborn piglets, necessitating alternative immunization strategies.

**Figure 7:**
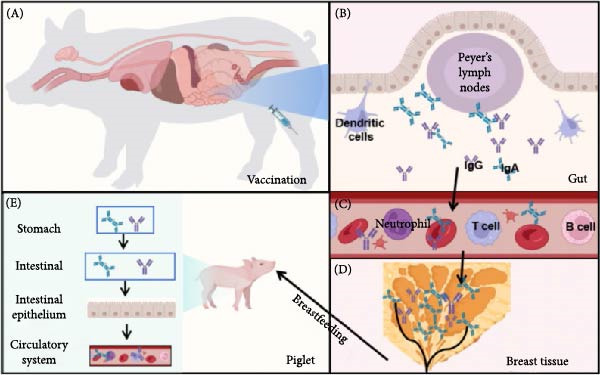
Gut–mammary axis pathway. This diagram illustrates the key mucosal immune principle of the “gut–mammary axis.” The core process is as follows: (A) Immune sensing and initiation: The mother is vaccinated via oral or injection routes. The vaccine antigens enter the intestine, where they are recognized by Peyer’s patch lymph nodes, initiating a specific immune response. (B) Cell activation and differentiation: Within the Peyer’s patch lymph nodes, T‐cells and B‐cells are activated. With the assistance of T‐cells, B‐cells differentiate into plasmablast precursors capable of producing IgA. (C) Cell migration and homing: These antigen‐specific lymphocytes (primarily IgA plasmablast precursors) enter the circulatory system and specifically migrate (home) to distant mucosal effector sites, such as mammary tissues. (D) Antibody secretion: Within mammary tissues, plasmablast precursors further mature and secrete large amounts of vaccine‐specific IgA antibodies. (E) Passive immunity: The antibodies are ingested by newborn piglets through maternal milk, forming an immune barrier on the surface of their intestinal mucosa, thereby providing essential passive immune protection.

Maternal vaccination of pregnant sows has emerged as the most effective intervention for mitigating PEDV transmission and improving survival rates in suckling piglets. This approach capitalizes on the biological mechanism of lactogenic immunity, whereby immunoglobulins secreted in colostrum and milk provide passive protection during early life stages. During lactation, sows transfer critical immune components, including sIgA, IgG, and IgM, through their breast milk, with these immunoglobulins demonstrating efficacy against enteric pathogens such as PEDV and TGEV [134, 135]. The mucosal protective role of PEDV‐specific IgA warrants special emphasis: through antigen binding at intestinal surfaces, it inhibits viral attachment and subsequent invasion of epithelial cells. This protective mechanism is facilitated by the gut–mammary gland sIgA axis, which translocates maternally derived IgA antibodies to mammary secretions following prepartum immunization (Figure 7).

The temporal dynamics of immunoglobulin absorption present critical considerations for optimal protection. Although colostrum contains elevated concentrations of IgG and IgA, neonatal intestinal permeability to these macromolecules persists only during the first 24 h postpartum, a biological phenomenon termed “intestinal closure” [134]. Maximum immunoglobulin transfer occurs within the initial 2–6 h postfarrowing, as colostral IgG and IgA concentrations peak around 2 h and maintain plateau levels for approximately 6 h before rapidly decreasing [136]. This narrow absorption window underscores the operational imperative for ensuring timely colostrum intake, particularly within the first 6 h of life. Strategic enhancement of colostral IgA production through maternal immunization could therefore serve as a pivotal management tool for maintaining intestinal health in piglet populations during PEDV outbreaks.

### 4.5. Standardize Efficacy Metrics

To further advance the development of PEDV and TGEV vaccines, it is necessary to establish and widely adopt a standardized efficacy evaluation system, which facilitates the comparison between different vaccine platforms. In the target animals, systematic evaluation should be conducted using the following quantifiable indicators: (1) for humoral immunity, virus‐specific IgG/IgA levels in serum can be quantitatively measured using ELISA, and neutralizing antibody titers in serum can be determined through virus neutralization tests (VNTs) or alternative methods such as pseudo VNTs (pVNT), for example, VNT_50_ [137, 138]; (2) for mucosal immunity, sIgA levels and their activity in intestinal lavage fluid or feces can be detected using ELISA and neutralization tests, as sIgA serves as the first line of defense against viral colonization and invasion in the intestine [139, 140]; (3) for cellular immunity, the magnitude and functional diversity of specific CD4^+^ and CD8^+^ T‐cell responses can be quantitatively analyzed using IFN‐*γ* ELISpot or intracellular cytokine staining combined with flow cytometry, which are crucial for viral clearance and cross‐protection [141, 142]; (4) for clinical symptoms, key indicators include animal survival/mortality rates, incidence and duration of severe diarrhea (assessed using a scoring system where: 0 = normal, 1 = soft stool, 2 = watery diarrhea, and 3 = severe watery diarrhea), vomiting frequency, body weight changes reflecting growth performance (such as average daily weight gain), and mental and dehydration status [143, 144]; (5) for virological assessment, focus on viral shedding and tissue damage. Viral loads in feces can be quantitatively measured by real‐time RT‐PCR, and virus isolation can confirm the presence of infectious viruses. Additionally, at the experimental endpoint, viral loads in intestinal tissues can be analyzed through necropsy, histopathological examination, and measurement of the villus height to crypt depth (VH/CD) ratio, to precisely assess the vaccine’s protective effect against typical intestinal lesions caused by the virus. An effective vaccine should maximally maintain the structural integrity of the intestinal mucosa [145, 146].

## 5. Discussion

### 5.1. Limitations of Conventional Vaccines and Viral Evolutionary Challenges

The global swine industry continues to face serious threats from porcine enteric CoVs, such as PEDV and TGEV. These viruses are characterized by significant genetic variability and antigenic drift, underscoring the urgent need for innovative vaccination strategies [147].The emergence of novel variants is driven by the interplay between intrinsic molecular features and external selection pressures. The high genetic diversity observed in these viruses fundamentally stems from the inherent lack of proofreading activity in their RdRp, which results in the continuous generation of diverse random mutations during each replication cycle [148]. Meanwhile, this characteristic also results in a high nucleotide misincorporation rate, which remains on the order of 10^−3^–10^−5^ per replication cycle. Intrinsic molecular characteristics of the virus facilitate the generation and maintenance of diverse viral quasispecies, enabling rapid adaptation to immunological and environmental selection pressures. Beyond the virus’s inherent genetic traits, external selective pressures, particularly immune pressure present in high‐density swine farming operations, also promote the generation of genetic diversity. Moreover, suboptimal immune conditions, whether induced by previous infection or imperfect vaccination, selectively favor the survival of viral mutants exhibiting altered antigenic properties. Furthermore, modern intensive farming practices significantly facilitate the continuous transmission of viruses and recombination among variant strains, thereby further promoting the expansion of viral diversity. Consequently, the evolution of these viruses is not a stochastic process, but rather a directed one arising from the ongoing coevolution between host and pathogen in an immune‐pressured environment.

The primary limitation of current vaccines against PEDV and TGEV lies in the prevalent issue of “evolutionary mismatch.” Most conventional vaccines, particularly inactivated whole‐virus formulations, are developed based on historical strains. However, due to antigenic drift caused by ongoing viral mutations, the immune responses elicited by these vaccines often fail to confer effective protection. Many of these mutations are notably concentrated within the NTD of the viral S protein. This situation creates a “selection‐evasion cycle” wherein vaccine‐induced immune responses exert directional selective pressure on highly mutable antigenic sites, thereby rapidly enriching for neutralization‐escape variants [6, 149–151]. This challenge is further exacerbated by the virus’s error‐prone replication machinery and recombination capability, which drive the continual emergence of novel quasispecies. Moreover, a key limitation of existing vaccines is their inability to elicit effective mucosal immunity alongside systemic responses: although parenteral administration (e.g., intramuscular injection) effectively induces systemic IgG antibodies, it fails to elicit robust mucosal immunity in the intestinal tract—the primary site of viral entry and replication [152]. Consequently, even vaccinated animals may exhibit suboptimal protection against intestinal infection, allowing the virus to replicate, shed, and transmit. This deficiency not only contributes to breakthrough infections but also sustains viral circulation and evolution under partial immune pressure. Thus, the development of next‐generation vaccines should prioritize targeting conserved epitopes and employing mucosal delivery platforms to address these two critical limitations [153].

### 5.2. Next‐Generation Vaccine Strategies for Broad Protection

To address the persistent evolution of PEDV and TGEV strains and overcome the inherent limitations of conventional vaccines, next‐generation vaccine strategies are increasingly focused on targeting “structurally conserved functional domains that are refractory to viral evolution.” This approach centers on viral conserved regions—such as fusion motifs in the S2 subunit of the S protein or functionally constrained residues within the RBD—to minimize immune escape and ensure vaccine resilience against viral diversification. These conserved epitopes can be engineered into chimeric or consensus‐sequence immunogens and efficiently presented on self‐assembling NP platforms. By leveraging multivalent display, such platforms not only broaden antigen coverage but also enhance immunogenic potency. Consequently, the rational design of novel vaccines should integrate advancements in mucosal immunology, nucleic acid–based vaccine platforms, and nanotechnology to drive the development of broadly protective vaccines. For instance, next‐generation mucosal vaccines, such as orally delivered engineered PEDV strains, aim to mimic natural infection by inducing sustained intestinal sIgA production and tissue‐resident immunity, thereby fortifying frontline defenses at viral entry portals [154]. Concurrently, saRNA vaccines demonstrate unprecedented neutralizing breadth against heterologous variants, leveraging their enhanced antigen expression kinetics to outpace conventional mRNA formats [155]. Nanocarrier engineering further revolutionizes antigen delivery through thermostable, size‐optimized particulate systems that potentiate dendritic cell activation and cross‐presentation. These multidisciplinary advances herald a paradigm shift toward variant‐resilient universal coronavirus vaccines, integrating rational antigen design with mucosal‐primed and systemic immunity. Future efforts must prioritize scalable production frameworks and cross‐protective epitope targeting to address both endemic and emergent strains, ultimately transforming pandemic preparedness in swine populations [156].

### 5.3. Future Directions

#### 5.3.1. Commercialization Challenges

The commercialization of vaccines for PEDV and TGEV faces multiple obstacles related to production, cost, and efficacy. During the production process, conventional vaccines suffer from issues such as long production cycles and the risk of reversion [48]; whereas novel vaccines, such as VLP vaccines, are prone to aggregation during purification and have low scalable yields, mRNA vaccines have complex and costly LNP preparation, and transgenic plant vaccines experience significant reduction in effective dosage due to degradation in the gastrointestinal tract after oral administration [76, 90]. Furthermore, there is currently a lack of standardized potency indicators for novel vaccines, making it difficult to compare different products and consequently slowing down the commercialization approval process. Therefore, for vaccines to successfully achieve commercialization, it is necessary to overcome multiple constraints related to production, cost, efficacy, and application scenarios.

#### 5.3.2. Pathways for Novel Platforms

The evaluation of novel vaccine platforms mainly focuses on three core aspects: safety, high efficacy, and stability of the production process. Regarding safety regulation, the main focus is on the control of potential risks and long‐term monitoring. Standardized safety assessment guidelines can be formulated to evaluate long‐term safety, genetic stability, and environmental impact. For example, in risk assessment of nucleic acid vaccines, DNA vaccines need to verify the “risk of genomic integration,” mRNA vaccines need to assess the toxicity of LNPs, and saRNA/circRNA vaccines need to verify replication safety [157]. For efficacy regulation, it is necessary to establish quantitative indicator standards. Regulatory authorities should mandate the following indicators: in terms of humoral immunity, including serum neutralizing antibody titers and intestinal sIgA levels; in terms of cellular immunity, including the proportion of IFN‐*γ* ^+^ CD8^+^ T‐cells and CTL activity; and in terms of virological indicators, including reduction in fecal viral load and the VH/CD ratio in the intestine [6, 140, 141, 144]. Regarding manufacturing regulation, the focus should be on process validation, stability testing, and environmental safety.

#### 5.3.3. Integration of Multidisciplinary Approaches

First, drive the precise design of antigens through AI and bioinformatics. By utilizing high‐throughput sequencing and phylogenetic analysis to track viral mutations in real time, and employing bioinformatics tools along with AI algorithms, it is possible to accurately predict changes in viral immunogenicity and conserved sites, thereby rationally designing immunogens with broad potential [158, 159]. Second, achieve precise antigen delivery through structural biology and nanotechnology. Structural biology methods guide antigen engineering—for example, introducing specific site mutations to significantly enhance the thermal stability of the S protein; subsequently, NPs compatible with the antigen’s size and structure can be designed to present its native conformation, ensuring delivery efficiency and effective immune recognition [160, 161]. Finally, precisely induce immune responses through microbiology and mucosal immunology. Using microbiological techniques to construct vaccines based on *Salmonella* or probiotic vectors, and leveraging in‐depth understanding of mucosal immune pathways, strategies such as incorporating M cell‐targeting peptides can significantly enhance antigen enrichment in intestinal lymphoid tissues, thereby efficiently inducing mucosal sIgA responses and establishing the first line of defense [162, 163].

## Conflicts of Interest

The authors declare no conflicts of interest.

## Author Contributions

**Xiaojun Hu, Entao Li, Zhenshan Wang, and Shen Wang:** writing – review and editing, writing – original draft, visualization, validation, project administration, methodology, investigation, conceptualization. **Hongyu Sun:** investigation. **Na Feng, Xianzhu Xia, Guixue Hu, and Feihu Yan:** validation, supervision, methodology, investigation, conceptualization.

## Funding

This work was supported by the Jilin Provincial Natural Science Foundation Project (Grant 20230101257JC), the Hunan Province Natural Science Foundation of China (Grant 2023JJ50141), the Health Commission of Hunan Province (Grant 202211004302), and the National Natural Science Foundation Project (Grant 32072857).

## Data Availability

The data that support the findings of this study are available from the corresponding author upon reasonable request.
